# Inoculum and inoculation techniques: key steps in studying pathogenicity and resistance to Sclerotinia stem rot in oilseed rape

**DOI:** 10.3389/fpls.2025.1610049

**Published:** 2025-07-18

**Authors:** Nazanin Zamani-Noor, Malgorzata Jedryczka

**Affiliations:** ^1^ Julius Kühn-Institute (JKI), Institute for Plant Protection in Field Crops and Grassland, Braunschweig, Germany; ^2^ Pathogen Genetics and Plant Resistance Team, Institute of Plant Genetics, Polish Academy of Sciences, Poznań, Poland

**Keywords:** *Brassica napus*, *Sclerotinia sclerotiorum*, cotyledon inoculation, intact stem inoculation, detached leaf assay, detached stem assay, Sclerotinia disease incidence and severity

## Abstract

*Sclerotinia sclerotiorum* is a soilborne and necrotrophic fungal pathogen that causes substantial yield and economic losses in oilseed rape cultivation worldwide. To date, no immune oilseed rape germplasm has been identified, posing a major challenge for breeding resistance to Sclerotinia stem rot. Developing reliable assessment techniques to evaluate oilseed rape resistance to the disease is a critical step in investigating genetic control and producing resistant cultivars. Extensive evaluations of oilseed rape genotypes have been conducted under both field and controlled conditions to assess resistance to Sclerotinia stem rot. Most inoculation techniques employ mycelium or mycelium-colonized substrates such as agar plugs, cereal grains, toothpicks, or petals. The use of ascospores as inoculum has been less common, despite their important role in the natural infection cycle. Several inoculation methodologies for controlled environments have been developed and evaluated for screening oilseed rape germplasm, including detached leaf/stem assays, intact leaf assays, cotyledon screening, as well as petiole and leaf axil inoculation. In parallel, several methods have been developed to assess Sclerotinia resistance under field conditions, such as intact stem inoculation at the flowering or maturity stage using *S. sclerotiorum*-infested toothpick, spraying ascospore suspensions onto plants, and spreading *S. sclerotiorum*-infested wheat grains. This review explores the suitability of various *S. sclerotiorum* inoculum types and evaluates the most common inoculation techniques for effective identification of susceptible and resistant oilseed rape genotypes to Sclerotinia stem rot.

## 1 Introduction

*Sclerotinia sclerotiorum* (Lib.) de Bary is a soilborne, necrotrophic, and omnivorous fungal plant pathogen capable of infecting more than 450 plant species, including oilseed rape (Boland and Hall, 1994; Bolton et al., 2006; Peltier et al., 2012; Ding et al., 2021; Newman and Derbyshire, 2020; O’Sullivan et al., 2021; Zamani-Noor et al., 2024a, 2025). The pathogen is responsible for causing stem rot or white mold in oilseed rape, leading to significant reductions in seed number, weight, and overall yield quality (Bolton et al., 2006; Buchwaldt, 2007; Saharan and Mehta, 2008; Paulitz et al., 2015; Derbyshire and Denton-Giles, 2016). Sclerotia are the primary survival forms of *S. sclerotiorum* (Coley-Smith and Cooke, 1971; Willetts et al., 1980; Smolinska and Kowalska, 2018). These structures account for approximately 90% of the pathogen’s life cycle, during which it remains in a dormant state. Sclerotia develop from mycelia present on or within infected plant tissues (Adams and Ayers, 1979; Young and Werner, 2012).

Plant infection occurs through the infrequent myceliogenic germination of sclerotia (Lane et al., 2019) or, more commonly, through ascospores released from apothecia during the carpogenic germination of sclerotia. If carpogenic germination coincides with the flowering stage of oilseed rape (BBCH 60-69) under favorable weather conditions, disease symptoms can develop rapidly. Sclerotia produce trumpet-shaped fruiting bodies known as apothecia (Willetts et al., 1980; Clarkson et al., 2014), with a single apothecium capable of dispersing up to 60 million airborne ascospores, which are spread by air currents and infect aerial plant parts (Clarkson et al., 2014; Willbur et al., 2019). Ascospores require senescent tissues to infect oilseed rape plants, typically consisting of fallen petals on leaves or within leaf axils (Sharma et al., 2015; Derbyshire and Denton-Giles, 2016). These petals serve as an energy source during infection, creating optimal conditions for ascospore germination and penetration of aerial plant parts covered with ‘mycelial cushions’ (Sharma et al., 2015; Derbyshire and Denton-Giles, 2016; Zamani-Noor, 2021). Following infection, characteristic *Sclerotinia* lesions initially appear on leaves and leaf axils as water-soaked spots or lesions with a pale greyish-white or brownish-white coloration, resulting from cell death and pathogen pectolytic enzyme-activity (Xu et al., 2018; McCaghey et al., 2019; Wang et al., 2019). These water-soaked lesions, particularly prominent at the nodes, tend to extend along the stem and rapidly encircle it above and below the affected nodes. Infected leaves may detach and become lodged further down the canopy, thereby facilitating the spread of infection to neighboring plants (Kamal et al., 2016; Willbur et al., 2019). The risk of infection is increased by semi-cold to warm and humid conditions during flowering, which are common in many oilseed rape production areas in Europe (Peltier et al., 2012). Consequently, the incidence and severity of the disease fluctuate annually and regionally (Barbetti and You, 2014). Yield losses caused by Sclerotinia stem rot range from 10% to 80%, depending on environmental conditions and severity of infection, with significant outbreaks reported worldwide in major oilseed rape-producing regions (Twengström et al., 1998; Derbyshire and Denton-Giles, 2016). In addition to direct yield losses, the disease adversely affects seed quality and oil content, reducing its economic value.

No sources of immunity to *S. sclerotiorum* have been found thus far in the family Brassicaceae. Consequently, numerous studies have focused on identifying genes involved in fungal development and pathogenesis (Li et al, 2004) or analyzing genetic loci associated with partial resistance in oilseed rape to the pathogen (Wu et al., 2016a, b). To date, various control strategies have been used to control Sclerotinia stem rot, including cultural practices, fungicide application, and varietal resistance (Bardin and Huang, 2001; Peltier et al., 2012; Tian et al., 2020; O’Sullivan et al., 2021; Zamani-Noor et al., 2024b). However, the persistent nature of sclerotia and the broad host range of this pathogen often limit the effectiveness of agricultural practices such as crop rotation (Peltier et al., 2012). Moreover, managing the disease through chemical control is often challenging due to difficulties in synchronizing fungicide applications with ascospore release (Bolton et al., 2006; Paulitz et al., 2015). Therefore, research on host resistance continues to gain importance as the most economically viable and sustainable approach within integrated pest management strategies for controlling Sclerotinia stem rot (Sharma et al., 2015; Denton-Giles et al., 2018; Wang et al., 2019).

This review provides an overview of various *S. sclerotiorum* inoculum sources and evaluates a range of inoculation techniques to effectively identify susceptible and resistant oilseed rape genotypes against Sclerotinia stem rot.

### 1.1 Plant inoculation – aims and strategies

Inoculation is the process of intentional introduction of viable plant pathogens into a host using a suitable medium to promote their growth, propagation, and colonization, ultimately leading to the development of disease symptoms. The main reason for inoculation is to evaluate the effects of a specific pathogen on a particular plant species and to assess the crop’s reaction and disease resistance. This approach is important for understanding the genetic mechanisms underlying host resistance to pathogens and developing resistant plant cultivars. Artificial inoculation is particularly useful when natural infection is not feasible, as it facilitates genotypic host differentiation and reduces the influence of plant morphological traits that may contribute to disease avoidance (Bradley et al., 2006; Li et al., 2007; Gupta et al., 2020).

Assessing resistance to Sclerotinia stem rot presents considerable challenges when relying solely on natural infection, primarily due to significant fluctuations in disease incidence and severity across different regions and time (Li et al., 2006; Zamani-Noor et al., 2025). These variations are strongly influenced by environmental parameters, such as temperature and precipitation, which are difficult to control (Li et al., 2007; Gupta et al., 2020). Additionally, Sclerotinia stem rot may be completely absent under specific environmental conditions (Gupta et al., 2020). Achieving consistent differentiation in levels of resistance to Sclerotinia stem rot requires the adoption of inoculation methods (Bradley et al., 2006; Gupta et al., 2020). Accordingly, various inoculation techniques have been developed and implemented to facilitate the evaluation of disease progression and to screen breeding materials for resistance against Sclerotinia stem rot. Identifying appropriate inoculum types is equally important, as it helps to determine the most effective approach for disease prevention. Different inoculum sources have been utilized in studies on Sclerotinia stem rot in oilseed rape under both field and controlled greenhouse conditions (Li et al., 2007). Consequently, the techniques used for inoculation vary depending on the specific type of inoculum employed.

The preparation of inoculum begins with the proper isolation, handling and storage of pure *S. sclerotiorum* cultures, preferably derived from the plant species to be inoculated. Although there is no direct evidence of the presence of physiological races in *S. sclerotiorum*, it is assumed that isolates obtained from the same host plant are best adapted and do not require an intermediate step of primary inoculation and re-isolation from oilseed rape. However, to preserve the aggressiveness and high infectivity of the isolate(s) used for inoculation, classical plant pathology textbooks recommend regular revival, ideally on an annual or biannual basis (Raimbault and Alazard, 1980; Nakasone et al., 2004). The simplest method for storing re-isolated strains involves keeping sclerotia at 4°C (Zamani-Noor, 2021). Alternatively, the pathogen can be also stored as mycelium at –80°C in cryo-freezer solution composed of 5% skimmed milk and 20% glycerol (Gyawali et al., 2016).

## 2 Inoculum types

**Figure 1:**
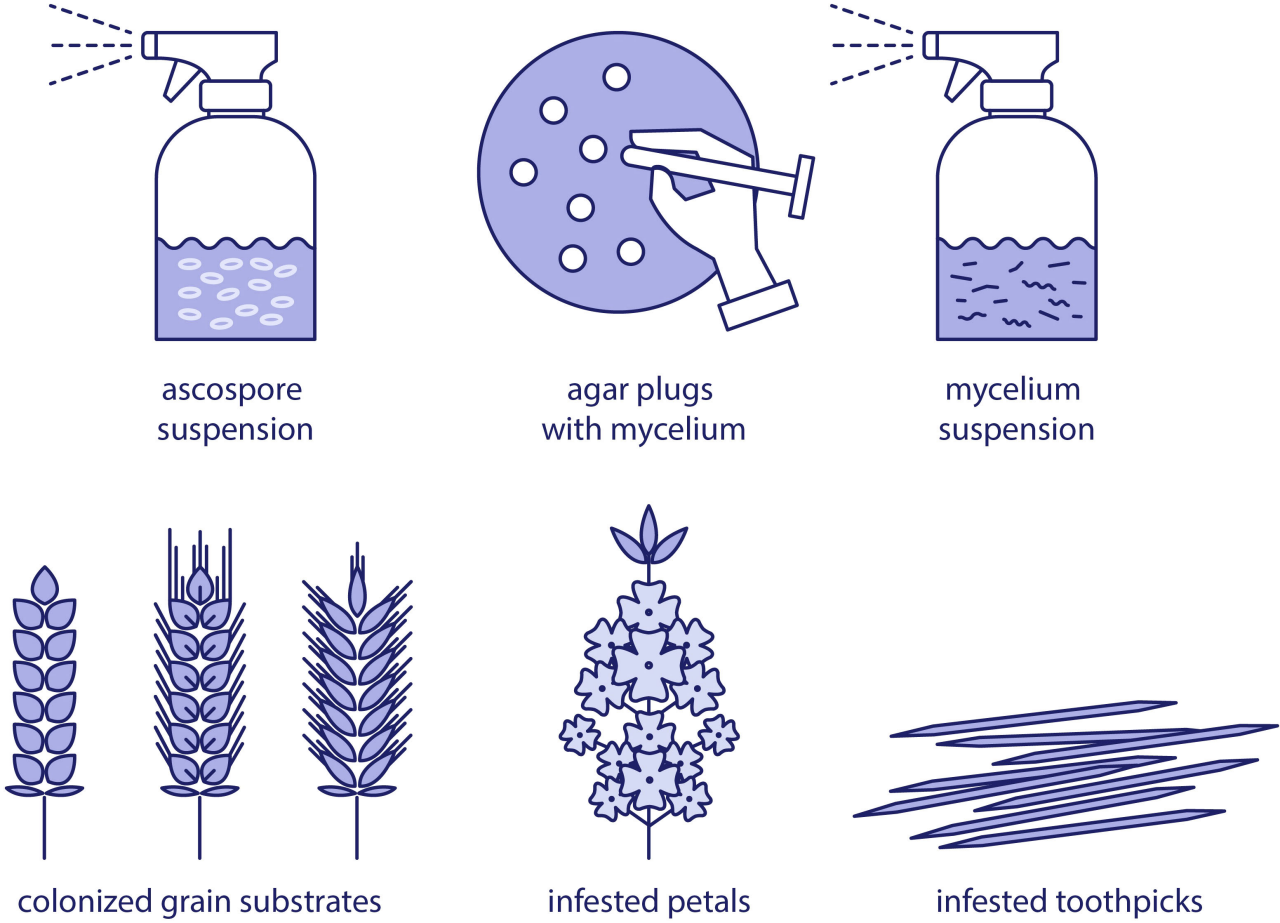
Overview of inoculum types of *Sclerotinia sclerotiorum* in plant pathology research.

There are several well-established methods for the preparation of *S. sclerotiorum* inoculum, each tailored to specific research objectives and experimental setups (**Figure 1**). The choice of inoculum type significantly influences the outcome of pathogenicity assays, disease epidemiology studies, and fungicide efficacy tests.

### 2.1 Ascospore suspension

Considering the life cycle of the fungus *S. sclerotiorum*, breeders and researchers aiming to identify resistance sources and employing inoculation techniques should focus on infecting plants with ascospores. This represents the primary and most natural plant infection pathway for the pathogen. For this reason, the concentration of ascospores released from sclerotia has been extensively studied. Research has indicated a clear relationship between the appearances of airborne ascospores and weather conditions affecting sclerotia development (Rogers et al., 2009). Consequently, monitoring sclerotial development and systematically observing apothecia formation have become an indicator for determining the timing of plant infection (Clarkson et al., 2004; Reich and Chatterton, 2022; Zamani-Noor et al., 2024a). Studies by Jamaux and Spire (1994) conclusively demonstrated that pathogenesis involving ascospore adhesion, germination, and host-plant penetration occurs exclusively when ascospores infect delicate plant petals, while they are unable to infect leaves. This underscores the critical role of petals in the pathogen’s development cycle. Further research has identified the transcription factor SsNsd1 as a key regulator in transitioning between the asexual and sexual phases of the pathogen’s life cycle and in appressorium formation (Li et al., 2017). The absence of this transcription factor results in the inhibition of its development, ultimately preventing the fungus from infecting the host plant.

The production of ascospores in the laboratory is a time-consuming procedure. The most efficient method for generating clean sclerotia involves culturing them on carrot agar (Freeman et al., 2002). Subsequently, the sclerotia should be buried 2–3 cm deep in a pot filled with vermiculite or perlite and placed in a tray with water maintained just below the level of the sclerotia. To preserve moisture, the trays must be covered and stored in a cold room at 5-10°C for approximately 2–3 months. After vernalization, the pots containing the sclerotia should be moved to room temperature, underexposed to near-UV light and kept moist. Once mature apothecia emerge, the tray lids should be opened, and the resulting ascospore clouds collected onto nitrocellulose filters. Freeman et al. (2002) have also described a PCR-based assay for detecting *Sclerotinia* ascospores using primer sequences targeting rDNA ITS fragments. The study has demonstrated that species-specific detection of *S. sclerotiorum* ascospores is possible using common air-sampling devices, such as Burkard spore traps.

### 2.2 Mycelium suspension

This form of inoculum utilizes a suspension of mycelium in water as a source of plant infection. Its ease is attributed to the rapid growth of *S. sclerotiorum* and the method’s ability to accurately quantify the initial concentrations required for inoculation.

The preparation of a mycelial suspension can follow the protocol describe by Garg et al. (2008), involving excising agar plug discs (5 mm) from the actively growing margins of 3-day-old *S. sclerotiorum* colonies cultivated on potato dextrose agar (PDA) at 20°C. These discs are subsequently transferred to 250 mL flasks containing 75 mL of sterilized liquid medium composed of potato dextrose broth (24 g), peptone (10 g), and distilled water (1 L). The flasks are placed on a platform shaker operating at 120 rotations per minute (rpm) for three days. Following this incubation period, *S. sclerotiorum* colonies are harvested and rinsed twice with sterilized deionized water. The resulting fungal mats are then transferred to 125 mL of the same liquid medium and macerated using a blender for 3 minutes. The resulting suspension of mycelial fragments is filtered through four layers of cheesecloth, and its concentration is adjusted to 1×10^4^ fragments mL^−1^ using a hemocytometer.

Mycelial suspension can be directly applied to inoculate oilseed rape plants under moist weather conditions. However, it is most commonly used as an intermediate step to prepare other forms of inoculum, mainly for infecting cereal grains, toothpicks or wooden clothespins, which are subsequently utilized for plant inoculation.

### 2.3 Agar plugs with mycelium

Agar plugs are among the most commonly used methods for inoculating oilseed rape plants (Li et al., 2004; Buchwaldt et al., 2005). Due to the rapid growth rate of *S. sclerotiorum*, the fungus is typically cultured on 9-cm Petri dishes containing PDA, baked bean agar (BBA) or other laboratory media (Kamal et al., 2015). Gyawali et al. (2016) used a modified glucose medium described by Cruickshank (1983), consisting of 20 g of glucose, 3 g of malic acid, 2 g of NH_4_NO_3_, 1 g of KH_2_PO_4_, 1 g of NaOH, 0.1 g of MgSO_4_7H_2_O, and 20 g of agar in 1 L of distilled water. Plates were inoculated with an agar plug, a filter paper disc covered with mycelium, or a sclerotium and incubated at room temperature, preferably under a day/night temperature regime (e.g., 22/18°C with a 16/8 h light/dark cycle). While most studies do not specify artificial lighting conditions, some experiments utilized white fluorescent bulbs to simulate day and night changes (Buchwaldt et al., 2005; Gyawali et al., 2016). Optimal inoculations are obtained using pathogen hyphae excised from actively growing culture margins (Zamani-Noor, 2021). The agar plugs are usually cut with a cork borer (5–7 mm diameter), and subsequent inoculation is performed by attaching the agar plug to the plant using a metal pin, aluminum foil or Parafilm.

However, this method of inoculation is time-consuming, as each plant genotype have to be inoculated manually and in multiple replicates. Additionally, it is important for the plants to be uniform and representative of the tested genotype. However, the preparation of agar plugs is quick and straightforward, making it accessible even to laboratories with minimal equipment. This simplicity and practicality have contributed to the method’s widespread use among researchers and breeders.

### 2.4 Cereal grain infested by *S. sclerotiorum*

The most common method for preparing *S. sclerotiorum* inoculum involves the use of cereal grains such as wheat, barley, rye or oats (Thomson and Kondra, 1983; Chaocai, 1995; Starzycka et al., 2000; Taylor et al., 2015). This form of inoculation is frequently employed in studies evaluating the efficacy of various fungicides (Mueller et al., 2002; Taylor et al., 2015; Zamani-Noor, 2021). The preference for this approach is connected with large sizes of individual plots usually used in such experiments. The extensive coverage of machinery arms or plant sprayers, whether mounted on small movable vehicles or large tractors, necessitates plot sizes of at least 10–20 m^2^. Given the need for 3-4 replicates per plot, these experiments pose a significant logistical challenge. This is particularly true as they often involve numerous treatments (active substances), multiple concentrations, and at least 2–3 cultivars differing in flowering time, growth type and plant height. Inoculation of such plots requires substantial quantities of inoculum and a high number of infected plants, which is not feasible with methods targeting individual plants. Moreover, plant yield is another important measure of the impact of the pesticide tested, and it requires large plot areas, often-harvested using combine harvesters.

The most comprehensive and up-to-date methodology for inoculating oilseed rape with cereal grains has been recently described by Zamani-Noor (2021). In this method, a mixture of *S. sclerotiorum* isolates stored as sclerotia were first cultured on PDA medium and incubated at room temperature for 5 days to obtain actively growing cultures. The experiment used 2-kg batches of wheat grains, which were hydrated with distilled water for 24 hours and then autoclaved twice in 60-L plastic bags. After cooling, the grains were inoculated with agar plugs, vigorously shaken and incubated for approx. 3 weeks until most of the grains were overgrown with *S. sclerotiorum* mycelium. During incubation, the grains were shaken 2–3 times per week to ensure even fungal growth. The final inoculum consisted of roughly ground mycelium-covered grains, prepared using a laboratory mill following a three-day air-drying period of the infected wheat.

This method is highly valuable; however, the success of inoculation strongly depends on weather conditions. To create favorable conditions for infection, leaf wetness and air humidity can be artificially increased—typically using irrigation systems in field trials or automatic humidifiers in controlled environments.

### 2.5 Petals infested with *S. sclerotiorum*

The use of petals infested with *S. sclerotiorum* mimics the natural infection process in the field due to the use of infection cushions, similarly to the strategy employed by *Botrytis cinerea* (Choquer et al., 2021). As regards *S. sclerotiorum*, these cushions are formed by fungal hyphae on senescent petals that fall from flowers and remain on wet leaves. Thatcher et al. (2017) used this method as an assay for screening flowering spring oilseed plants in a controlled environment, although it can also be widely applied in the field. However, as with the use of roughly ground cereal grains, the success of this inoculation method depends on the adherence of petals to leaves for a sufficient period, allowing the pathogen to grow from the petal to the leaf, penetrate it, or at least develop significantly on its surface. The production of large quantities of infected petals is more challenging than producing infected grains; the sterilization process damages the delicate petals causing them to wrinkle and become uneven, which interferes with their adherence to the leaf surface. On the other hand, the petals that are not surface-sterilized can quickly become overgrown with mycelium of other fast-growing, ubiquitous fungi, such as *Cladosporium* or *Alternaria* (Shelton et al., 2002; Fröhlich-Nowoisky et al., 2009). The issue is further exacerbated by recent global climate changes, which have extended and intensified the sporulation period of these fungi (Rodriguez-Rajo et al., 2005: Kasprzyk et al., 2016).

### 2.6 *S. sclerotiorum*-infested toothpick technique

The toothpick method is another commonly used approach to inoculate oilseed rape plants with *S. sclerotiorum*. This method involves inserting mycelium-covered toothpicks into the stem (Zhao and Meng, 2003). In a recent study, Zhang et al. (2022) described a procedure where toothpicks were placed in autoclavable bottles, boiled for 5–10 min in a 5% sucrose solution, and inoculated with *S. sclerotiorum* mycelia. After one week of incubation, the toothpicks, fully overgrown with mycelium, were inserted into the angle between the main and a side branch, approximately 25–30 cm above ground level. The inoculation site was then covered with a wet cotton pad to maintain moisture, and all inoculation points were sprayed with water twice a day for one week. This method is recognized as a local standard for oilseed rape inoculation (No. PSJG 1107.1-2009) in Shaanxi Province (Zhang et al., 2022). While the use of commercially available toothpicks facilitates standardization, the method is more suitable for spring oilseed rape due to its softer stems, as harder stalks of winter cultivars are more difficult to penetrate by softer toothpicks overgrown with mycelium. Moreover, the requirement to treat individual plants and maintain consistent moisture makes this method impractical for large-scale inoculation.

## 3 Inoculation techniques under controlled greenhouse conditions

**Figure 2:**
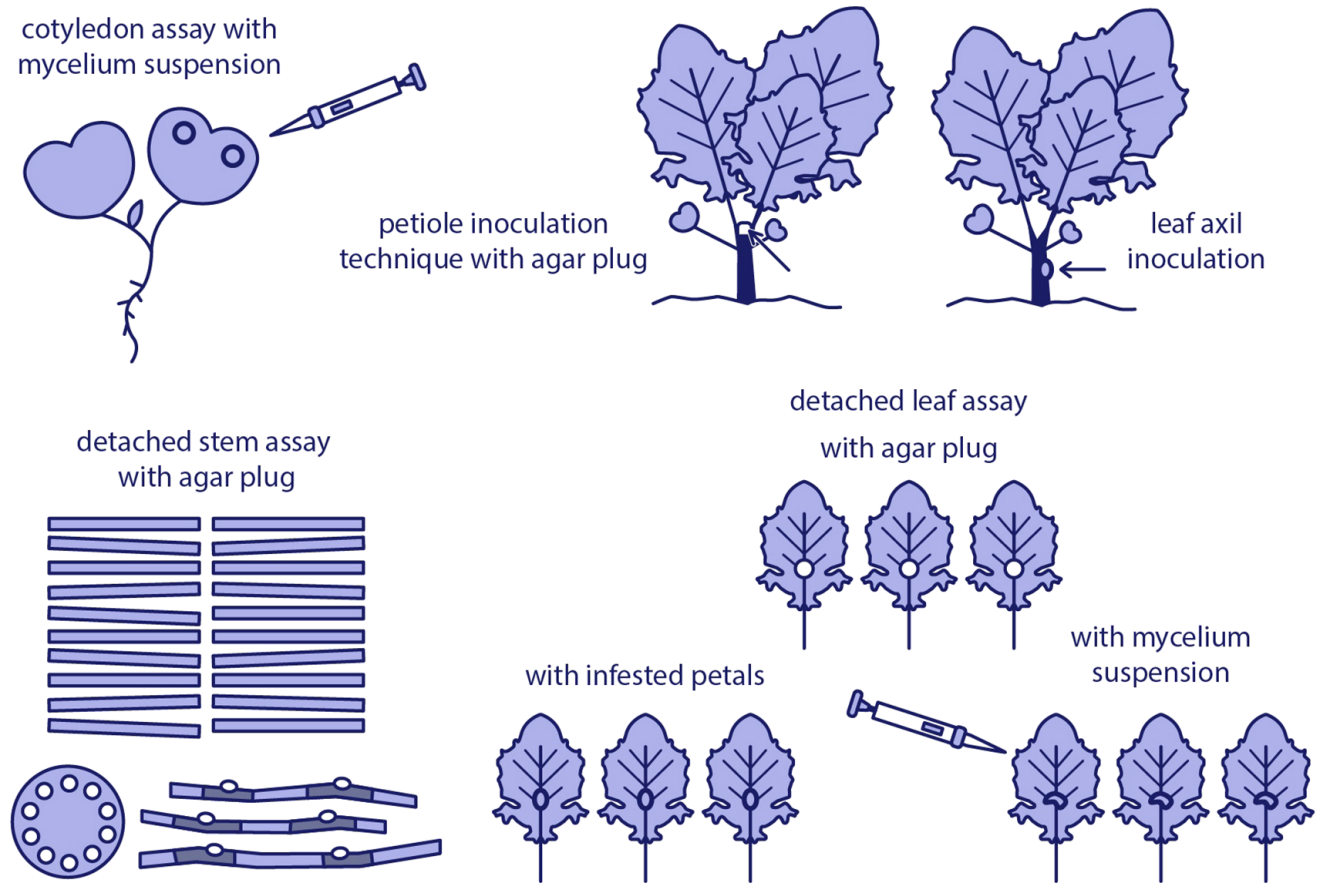
Overview of commonly used *Sclerotinia sclerotiorum* inoculation methods in oilseed rape under controlled conditions.

Greenhouse-based inoculation techniques offer a controlled environment to study the interaction between *S. sclerotiorum* and oilseed rape, allowing for consistent evaluation of pathogenicity and host resistance. Commonly used methods include cotyledon assays, whole-plant inoculation, and inoculation of detached leaves or stems, each offering specific advantages depending on the research objective (**Figure 2**).

### 3.1 Cotyledon assay

The method was performed by Garg et al. (2008) under controlled greenhouse conditions, maintaining a temperature of 18 ± 1°C during the day and 14 ± 1°C at night, with a light intensity of 150 µE m^−2^ s^−1^. Seeds of oilseed rape cultivars were sown in 14×7×5 cm trays, each containing eight cells filled with a soilless compost mixture. Three seeds of each genotype were sown per cell, and after germination, seedlings were thinned to a single seedling per cell. The seedlings were cultivated until their cotyledons were fully expanded at BBCH 10 (Garg et al., 2008).

For inoculation, four droplets of mycelial suspension (1×10^4^ fragments mL^−1^, 10 µl per droplet) were applied to each plant using a micropipette, with one drop placed on each cotyledon lobe. The mycelial suspension was regularly shaken during the inoculation process to ensure homogeneity. To maintain high humidity, a 2.5 cm-deep-water layer was kept at the base of the plastic containers. Additionally, a fine mist of water was sprayed over the cotyledons and the inside of the container lids. Following inoculation, the boxes were placed under the benches in a controlled environment room for two days, with a low light intensity of approximately 13 µE m^−2^ s^−1^ for 4 days until the disease assessment date. Typical hypersensitive and/or necrotic, and water-soaked lesions were evident within one to two days post-inoculation (dpi). At 4 dpi, box lids were removed, and lesion diameters were measured (mm).

The authors of the cotyledon assay concluded that this method was a reliable and reproducible approach for assessing the virulence of *S. sclerotiorum* in oilseed rape cultivars. They emphasized that using controlled environmental conditions and a standardized inoculation protocol enabled accurate assessment of fungal infection and host resistance. Furthermore, this technique is particularly effective for testing a large number of genotypes under controlled conditions, making it a valuable tool for studying disease resistance in crop breeding programs (Garg et al., 2008).

### 3.2 Petiole inoculation technique

The method was initially introduced by Del Rio et al. (2001) for soybean and later adapted for oilseed rape by Zhao et al. (2004), with further modifications by Bradley et al. (2006) to improve its efficacy. Briefly, oilseed rape seedlings were cultivated in 4×4 cm plastic pots (two plants per pot, three pots per replication) filled with potting mix. Inoculation of seedlings was performed at the four- to five-leaf stage. To inoculate, an 8-mm-thick plug of PDA and mycelium was obtained by inserting the open end of a 1000-μl pipette tip into the margin of a 3-day-old fungal colony. Before plant inoculation, pipette tips were preloaded and transported in sealed pipette tip boxes. Petioles were cut 2.5 cm from the main stem using a razor blade. The tapered end of each inoculum-filled pipette tip was sealed with an index finger, and the petiole was pushed through the agar plug inside the pipette tip until its end aligned with the end of the agar plug (Zhao et al., 2004). Plants were monitored over a 6-day period, and seedling mortality was recorded daily. Scoring was conducted on the day when irreversible wilting occurred or when a severe girdling lesion caused the stem above the lesion to collapse. The area under the disease progress curve (AUDPC) was calculated using the daily percentage of dead plants as the dependent variable and the six observation dates as the independent variable (Bradley et al., 2006).

In the articles referenced above, the authors have positively evaluated the petiole inoculation technique, emphasizing its effectiveness in assessing *S. sclerotiorum* virulence in oilseed rape. They underline that this technique provides a controlled and precise approach to plant inoculation, facilitating accurate monitoring of disease progression, including mortality and lesion development. Additionally, they highlight the utility of AUDPC as a comprehensive measure of disease severity over time, positioning the technique as a valuable tool for disease resistance research in crop breeding programs.

### 3.3 Leaf axil inoculation

This method was initially used to evaluate the aggressiveness of one *S. subarctica* isolate and 17 *S. sclerotiorum* isolates collected from various crop hosts on representative cultivars of *B. rapa* var. *rapa*, *B. oleracea* var. *italica*, and *B. napus* var. *napus* (Taylor et al., 2015). The inoculum of each isolate was produced by inoculating moist, sterilized wheat grains in Petri dishes with plugs cut from the actively growing edge of *S. sclerotiorum*/*S. subarctica* cultures on PDA medium and incubated at 20°C for 4 days. The colonized wheat grains were subsequently used to inoculate 7-week-old plants at the 7-9 leaf stage by placing a single wheat grain in the leaf axil of the third lowest leaf on each plant. To promote disease development, overhead mist irrigation was applied (5 minutes, three times during the dark period) to maintain a humid environment. *Sclerotinia* disease symptoms were recorded at 7, 10, 14, 17, 21, and 28 dpi. At each evaluation date, plants were rated for infection severity using a scale where 0 indicated no infection, 1 represented infection limited to the leaf petiole, and 2 denoted stem infection. The number of wilted leaves was also recorded, with the assessment standardized to monitor seven leaves per plant—four above the inoculation point and three below. Lesion length measurements were conducted exclusively for *B. oleracea* var. *italica*, as it was the only *Brassica* type with sufficient stem elongation.

The authors have declared that this method reliably differentiates the aggressiveness of various *S. sclerotiorum* isolates and effectively identifies resistance levels in Brassica species. They have emphasized the importance of employing aggressive, locally prevalent isolates in resistance screening to ensure the findings are both relevant and directly applicable to specific target regions. Furthermore, the authors recognize the challenges associated with variability between different screening methods and highlight the necessity of adopting complementary approaches to evaluate resistance at various stages of plant maturity.

### 3.4 Detached leaf assay

The detached leaf assay method was first successfully applied by Bailey (1987) to differentiate oilseed rape accessions for resistance to *S. sclerotiorum*. The method was later modified by Liu et al. (2005). In this approach, oilseed rape plants were cultivated under controlled greenhouse conditions until reaching the four-true-leaf stage. *S. sclerotiorum* mycelia were cultured on PDA, and agar discs were excised from the edges of actively growing colonies and placed onto detached leaves. All leaves were labeled and randomly arranged on wet gauze in containers covered with transparent polyethylene bags. The leaves in the containers were incubated at 20 ± 2°C in a dark room. Lesion sizes were measured in length and width 24 hours post-inoculation and at subsequent intervals. While this method has been widely utilized in various studies, perspectives on its effectiveness for evaluating *S. sclerotiorum* resistance in oilseed rape remain ambiguous. Bailey (1987) demonstrated the effectiveness of the detached leaf assay in differentiating resistance between oilseed rape accessions. Liu et al. (2005) subsequently refined the methodology, and Dong et al. (2008) reported consistent results, further supporting the utility of the technique. However, other studies have presented contrasting findings. For instance, Bradley et al. (2006) observed no differentiation among the tested cultivars and found a poor correlation with field data. Consequently, they argued that while leaf resistance may contribute to overall plant defense, stem resistance is likely of greater importance due to the more severe impact of stem infections on crop yield. In summary, although the detached leaf assay has shown promise in specific contexts, its predictive value for field resistance remains uncertain. This highlights the importance of exploring alternative resistance mechanisms, particularly those related to stem resistance.

### 3.5 Detached stem assay

The technique of inoculating detached stem under controlled environmental conditions was first reported by Mei et al. (2012) and subsequently applied extensively by Wu et al. (2016a, b). In their experiments, Brassica species were initially cultivated under natural field conditions. When the plants reached maturity (BBCH 75-85), approx. 30-cm-long stems were cut using a sharp knife, leaving a 10 cm stem portion above the ground. Two 30-cm segments were cut from the first two branches of the stem. The ends of the stem and branches were then wrapped in polyethylene film to preserve freshness. Subsequently, artificial wounds were made on the stem and branch segments using a 5-mm puncher, and 5-mm agar plugs, excised from the edge of a 3- to 4-day-old *S. sclerotiorum* culture grown on PDA medium, were placed over the wounds. The inoculation was carried out at two points along the 30-cm-long stems and branches, with a 10-cm spacing between the points, while the stem sections were inoculated at their midpoint. The inoculated stems and branches were put on a platform lined with moist towels and filter papers. This platform was assembled from boards measuring 2×2 m, on which a 0.5-m-high frame was placed. Following inoculation, the container was sealed with polyethylene, and the temperature was adjusted to 21°C. The length of the lesions (cm) and the perimeter of both stems and branches were measured three days after inoculation (Mei et al., 2012).

The authors have recommended this method for its reliability, adaptability, and efficiency in screening for resistance to *S. sclerotiorum*. They have emphasized its ability to produce consistent and reproducible results under controlled environmental conditions, minimizing variability compared to traditional field-based methods such as toothpick inoculation. Furthermore, the method’s scalability has been noted, as a single platform can accommodate over 150 stems, making it well suited for large-scale evaluations with limited resource requirements. The technique is particularly advantageous for resistance breeding programs, as it allows the use of branches for resistance screening while preserving the primary stems for seed production and further breeding works.

### 3.6 Intact leaf inoculation with mycelium suspension

The methodology was developed by Thatcher et al. (2017). Oilseed rape (spring *B. napus*) seeds were sown into pots (width: 18 cm, height: 40 cm) filled with a non-sterile potting mix soil blend and cultivated in a greenhouse from May to November. Day lengths ranged from 10 to 14 hours, and average day/night temperatures were maintained at 29°C/16.5°C. Pots were irrigated with approximately 300 mL of water three times per week. Aluminum-frame enclosure hoods wrapped in polyethylene plastic film were constructed to contain flowering oilseed rape-plants and placed on standard glasshouse trolleys. A plastic sheet under the trolleys helped maintain high humidity levels. Inoculations were performed on fully emerged vegetative leaves of 12-week-old non-flowering oilseed rape plants. The inoculum (a mycelium suspension) was prepared following the method of Garg et al. (2008), and its concentration was adjusted to 1 × 10^6^ fragments mL^−1^ based on hemocytometer counts. Following inoculation, the entire plant was covered with polythene film to preserve moisture. Disease assessments were conducted at 7 and 12 dpi by measuring lesion sizes.

The authors demonstrated that inoculating intact leaves with a mycelium suspension provided a controlled approach to studying *S. sclerotiorum* infections in oilseed rape. This method highlights the importance of consistent inoculum preparation and application to ensure reliable results while minimizing plant stress caused by the inoculation process. Focusing on intact leaves more accurately reproduced natural infection pathways compared to methods involving wounding, and provided important information on the early stages of disease development. However, the authors also acknowledged potential limitations, including variability in plant responses and challenges in accurately quantifying the inoculum reaching the site of infection.

### 3.7 Intact leaf inoculation with infested petals

Intact leaf inoculation using infested petals is another method of plant inoculation with *S. sclerotiorum* (Thatcher et al., 2017). Spring oilseed rape-plants were cultivated under the same conditions described for the intact leaf inoculation with a mycelial suspension. Petals collected from flowers were immersed in a mycelial suspension of 1 × 10^6^ fragments mL^−1^ and placed on the leaves. After inoculation, the entire plant was covered with polythene film to maintain humidity. Disease assessments were performed at 7 and 12 dpi by measuring lesion sizes.

The aforementioned inoculation technique aims to simulate a more natural infection route to assess resistance of spring oilseed rape-genotypes against Sclerotinia stem rot. The use of infected petals as an inoculum source can more accurately reflect how the pathogen spreads in the field, where infected plant debris, including petals, often serves as a reservoir for the pathogen. The authors discuss the advantages of this method, including its potential to provide more biologically relevant data on disease development compared to direct inoculation with a mycelial suspension. Additionally, this approach helps to better understand the role of senescing plant material in disease epidemiology. However, the method may have limitations, such as variability in petal size, infection levels, and the presence of other microorganisms on the petals, which can introduce inconsistencies in the inoculation process. Moreover, this method is more time-consuming and labor-intensive than direct inoculation with a mycelial suspension.

### 3.8 Intact stem inoculation

The method, developed by Li et al. (2004), involves cultivating oilseed rape plants under controlled greenhouse conditions with a 16:8 h day/night photoperiod and temperatures of 22°C/16°C in a humid environment. Stems of 8-week-old flowering plants were inoculated using a 3-mm diameter plug of *S. sclerotiorum* obtained from the actively growing margin of a colony cultured on PDA. The plug was secured to the stem surface using Parafilm and remained in place until lesion formation, which typically occurred within seven days. Disease incidence was evaluated by measuring the stem lesion length (cm) (Li et al., 2004).

The authors have emphasized that this approach demonstrates the importance of controlled inoculation conditions using standardized mycelial plugs to reduce variability and accurately assess oilseed rape plant responses to *S. sclerotiorum*. This method enables a targeted assessment of stem resistance mechanisms by directly exposing stem tissue to the pathogen. However, the authors acknowledge certain limitations of the method, such as its potential to oversimplify the natural infection process and its narrow scope, which may not account for other aspects of disease development beyond stem rot.

## 4 Inoculation techniques under field conditions

**Figure 3:**
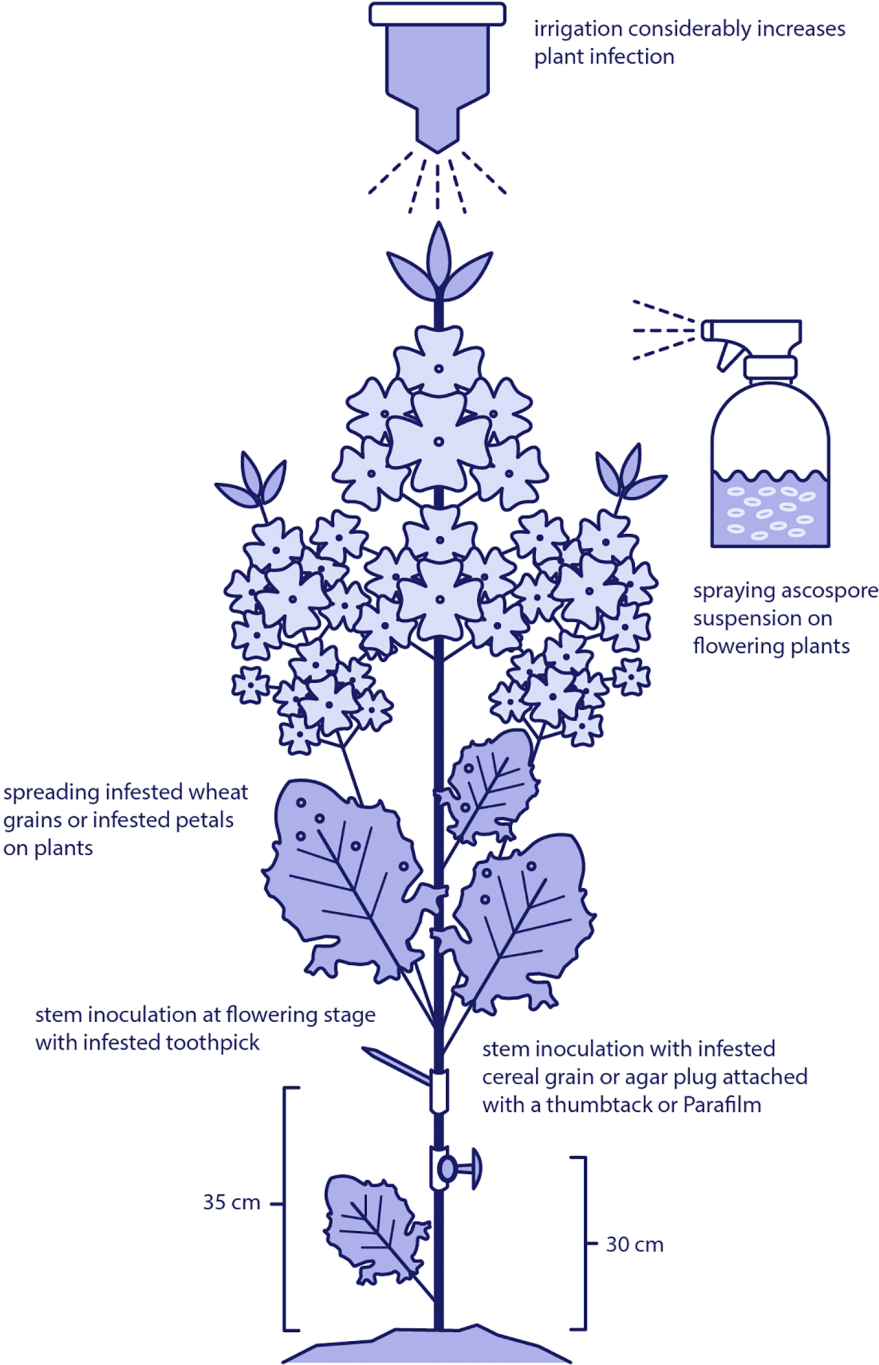
Field-based inoculation techniques used to study *Sclerotinia sclerotiorum* in oilseed rape. Methods include intact stem inoculation at the flowering stage using colonized grain, mycelial plugs, or infested toothpicks, as well as foliar application of ascospore suspensions or infested grain to simulate natural infection processes under field conditions.

Field-based inoculation methods are important for evaluating the pathogenicity of *S. sclerotiorum* and assessing host resistance under realistic agronomic conditions. These techniques aim to simulate natural infection processes while ensuring uniform disease pressure across experimental plots. Common approaches include intact stem inoculation at the flowering stage using colonized grain, mycelial plugs, or infested toothpicks, as well as spraying ascospore suspensions directly onto plant tissues (**Figure 3**).

Timing, environmental conditions, and inoculum density play essential roles in disease development and it have to be carefully standardized. Effective field inoculation facilitates reliable screening of genotypes and informs breeding programs targeting improved resistance to Sclerotinia stem rot in oilseed rape.

### 4.1 Intact stem inoculation at flowering stage

This inoculation technique was initially described by Li et al. (2004) and Buchwaldt et al. (2005), and subsequently modified by Li et al. (2007). Oilseed rape genotypes were sown in a nylon-mesh screen house in single 1-m-long rows, with 0.6-m spacing between them. Twenty seeds per genotype were sown, and seedlings were thinned to 12–13 plants per row two weeks after germination. Ten plants from each genotype were randomly selected and inoculated at the flowering stage, when 50% of the plants of each genotype had at least one opened flower. A single agar plug disc (5 mm in diameter) was used as an inoculum for each plant. The agar disc was cut from the actively growing margin of a 3-day-old colony cultured on a glucose-rich medium (peptone – 10 g, agar – 23 g, glucose – 20 g KH_2_PO_4_ – 0.5 g, H_2_O – 1 L, pH adjusted to 4.0 with HCl before autoclaving) and wrapped onto the first internode above the middle node of each stem using Parafilm. Plants were also irrigated by overhead sprinklers when natural rainfall was insufficient. Stem lesion length was measured three weeks post-inoculation using a ruler. Control plants were mock-inoculated with non-colonized agar plugs to verify that all infections observed were attributable to *S. sclerotiorum* isolate inoculation.

The extended time available for disease rating, lasting several weeks, allowed researchers to track disease progression from the flowering stage to plant maturity (Gyawali et al., 2016; Li et al., 2007). It was demonstrated that disease severity assessed 3 weeks after inoculation was more strongly associated with molecular markers for resistance compared to evaluations conducted one or two weeks after inoculation. This approach enabled the selection of oilseed rape phenotypes with consistent responses to inoculation from a larger collection of lines. Clear differentiation was achieved in lesion length and the extent of penetration into the vascular tissue.

The stem inoculation method has been widely adopted by research teams for screening various *Brassica* germplasm and crucifer species (Li et al., 2007; Garg et al., 2010; Navabi et al., 2010; Wu et al., 2013; Uloth et al., 2013). Researchers consistently emphasize that intact stem inoculation at the flowering stage under field conditions is a more realistic assessment of Sclerotinia stem rot development than greenhouse studies. However, the variability of field conditions, including fluctuating weather patterns and natural infection pressures, can significantly affect experimental outcomes. High humidity, often resulting from rainfall or mist irrigation, plays a critical role in facilitating pathogen development. When combined with favorable air temperatures, it creates optimal conditions for infection and disease progression. Conversely, suboptimal conditions, such as low humidity or temperatures outside the pathogen’s preferred range, can reduce disease severity, introducing variability in field trial results.

### 4.2 Stem inoculation with infested cereal grains attached with a thumbtack

Sterilized rye grains were placed on V2 medium overgrown with *S. sclerotiorum* mycelium (Starzycka et al., 2000). Although, the composition of the medium was not specified in the article, it corresponded to V8 agar juice described by Stevens (1974). The grains (approx. 100 per 9-cm Petri dish) were removed from the medium after 3 days and individually attached to sterile metal thumbtacks, one grain per pin. Inoculation was carried out at the flowering stage, 5-10 cm above the middle of the main stem, and inoculation site was covered with a strip of aluminum foil. Disease evaluation was conducted on 20 plants (replicates) for each genotype tested. Stem discoloration caused by the pathogen was measured at 14 and 46 dpi with 1 mm accuracy and compared to a standard cultivar arbitrarily selected by the breeding company. The results were converted to obtain the percentage of the standard designated as 100%.

The authors inoculated over 1500 plants and determined the method to be reliable and efficient. Both assessment dates (14 dpi and 46 dpi) revealed significant differences compared to the control (non-inoculated plants), as well as significant variations between the genotypes tested. However, the evaluation performed 7 weeks after inoculation was indicated as more suitable for disease resistance selection.

### 4.3 Intact stem inoculation at maturity stage with infested toothpick

The methodology was initially described by Zhao and Meng (2003). In summary, oilseed rape plants were sown and cultivated under natural field conditions, and approximately one month before harvest (BBCH 81-85), they were inoculated with an *S. sclerotiorum* isolate using the toothpick method. This process involved introducing sterilized toothpicks to PDA pathogen cultures for 48 hours, resulting in complete overgrowth of the toothpicks by the mycelium. The oilseed rape stems were then pierced with mycelium-covered toothpicks approximately 35 **cm** above ground level. The length of the lesions along the stems was subsequently measured five days after inoculation (Zhao and Meng, 2003).

The authors emphasized the reliability of the toothpick inoculation method for assessing stem resistance in mature oilseed rape plants under field conditions. They pointed out that this technique, which involves inserting pathogen-colonized toothpicks directly into plant stems, consistently produced measurable lesion lengths. This method was deemed highly effective in simulating natural infection processes and capturing phenotypic variations associated with resistance traits, making it a valuable approach for genetic mapping and breeding studies.

### 4.4 Spraying ascospore suspension on plots

Bradley et al. (2006) thoroughly described a spray inoculation method using ascospore suspensions of *S. sclerotiorum* on oilseed rape plants at 10-40% and 50-80% flowering stages. To ensure adequate disease pressure, the ascospore suspension (1 × 10^3^ ascospores/mL) was uniformly applied to the plots using a CO_2_-pressurized hand sprayer at 207 kPa and 131 L/ha. A mist-irrigation system with 1.2-m risers spaced 4.6 m apart moisturized the plots for 3 minutes every 30 minutes. This irrigation began just prior to the ascospore inoculation and continued until swathing, lasting approximately 5-6 weeks. Sclerotinia stem rot incidence was assessed by inspecting 50 adjacent plants in the central area of each plot. Plants were classified as infected if their main stem or branches displayed bleaching or shredding, accompanied by the presence of sclerotia.

The disease pressure during field trials showed year-to-year variability (Bradley et al., 2006). Despite misting the field plots and performing artificial inoculation with ascospores, certain environmental conditions conducive to infection, such as temperature and relative humidity, remained beyond control in outdoor settings. These uncontrollable factors directly influenced ascospore survival and infection dynamics, potentially contributing to the observed variations in disease pressure across locations and over time.

### 4.5 Spreading infested wheat grains in plots

A detailed description of the methodology was provided by Zamani-Noor (2021). Briefly, seeds of a winter oilseed rape cultivar were sown at a density of 60 seeds per m^2^ under natural field conditions. Plant inoculation was performed by manually spreading approximately 50 g of ground *Sclerotinia*-infested wheat seeds per m^2^ over the plant canopy at growth stages BBCH 64-65, when 40-50% of the flowers on the main raceme were open, and older petals were falling. Directly after inoculation, all plots were irrigated with 10 mm of water every other day until 10 dpi. The plots were visually assessed for Sclerotinia stem rot at growth stages BBCH 80-83, characterized by 10-30% of pods being ripe and seeds becoming dark and hard. One hundred randomly selected plants were collected from each plot and rated for Sclerotinia stem rot on a scale ranging from 0 to 3, where 0 indicated no symptoms, 1 denoted up to 25% of the stem circumference affected by lesions, 2 indicated between 25% and 50% of the stem circumference affected by lesions, and 3 signified nearly dead plants. The disease severity index was calculated based on the types of infection observed.

The author expressed a positive opinion about the inoculation method, stressing that artificial inoculation using ground grain inoculum infested with *Sclerotinia* mycelia, combined with mist irrigation, and successfully induced intense disease development. This approach allowed to obtain intense infections, with disease severity reaching up to 80%, and effectively simulated natural conditions for evaluating disease control strategies. However, it was noted that, in addition to the presence of inoculum and high humidity, air temperature was another critical factor influencing the development of Sclerotinia stem rot. The pathogen *S. sclerotiorum* has an optimal development temperature range of 12-20°C, with temperatures below 10°C or above 25°C significantly inhibiting disease progression.

## 5 Characterizing disease severity: approaches and considerations

The severity of the disease can be measured using several metrics, which may show varying correlations with the traits under study. These measurements are frequently represented as the area under the disease progress curve (AUDPC). The AUDPC is a quantitative measure of disease intensity over time, commonly applied in plant pathology to assess and compare disease severity between plant materials (Jackson, 2022). The trapezoidal method, based on a formula developed by Campbell and Madden (1990), is the most commonly calculation approach. The area under the injury progress curve (AUIPC) is another metric that incorporates plant developmental stages. However, comparisons of these metrics between experiments should be avoided due to the significant influence of environmental conditions, particularly weather, as well as subtle variations in inoculation procedures (e.g., differences in personnel experience and individual plant treatments) and the aggressiveness of the isolate(s) used.

### 5.1 Lesion length

Lesion length is a straightforward yet informative parameter for quantifying Sclerotinia stem rot, particularly in stem inoculation studies. Measurements are typically taken using rulers. However, lesion expansion may be asymmetrical, often extending further on one side of the inoculation site. In such cases, the mean lesion length, calculated using both the length and width of the lesion, might be a more representative indicator. Gyawali et al. (2016) reported strong correlation between disease severity and the response of molecular markers when lesion length was expressed as AUDPC.

### 5.2 Lesion area

Lesion area, expressed as a percentage of infected tissue or measured in square millimeters or centimeters, provides a more comprehensive and informative representation of disease severity compared to lesion length. This parameter can also be converted to an AUDPC value by plotting the percentage of infection over time and calculating the sum of the trapezoidal areas between time intervals (Jackson, 2022). These measurements are typically performed using electronic calipers. Lesion area assessment is applicable to both foliar and stem symptoms. When estimating lesion area on leaves, AUDPC is a semi-quantitative variable, but a precise value can also be obtained by applying professional scanners capable of differentiating colors.

### 5.3 Progression of symptoms into vascular tissues

Lesion length or area reflect the externally observable symptoms of disease severity on oilseed rape plants, whereas penetration into vascular tissues measures the extent of internal damage. This parameter is typically expressed as the percentage of soft and collapsed tissue within lesions (Gyawali et al., 2016; Denton-Giles et al., 2018). Accurate assessment requires considerable expertise, as scoring relies on various rating scales (e.g., 0-3, 1-9). In China, the assessment of Sclerotinia stem rot severity is carried out according to the local standard “Field resistance identification of *S. sclerotiorum* on oilseed rape” (No. DB51T 1035-2010). This standard categorizes plant responses into six classes: highly resistant (HR), moderately resistant (MR), resistant (R), moderately susceptible (MS), susceptible (S), and highly susceptible (HS) (Zhang et al., 2022).

## 6 Comparison and evaluation of inoculation methods

The inoculation methods described vary significantly due to adaptations to specific protocols and the availability of environmentally controlled chambers, greenhouse space, and field sites accessible to research teams studying with *S. sclerotiorum*. The key difference among these methods lies in inoculum preparation, which ranges from the relatively simple agar disc preparation (taking only a few days) to the more complex ascospore suspension preparation (requiring several months). Certain methods are better suited for detailed screening to identify resistance sources and evaluate progeny from crosses, while others are more appropriate for large-scale inoculations aimed at assessing the efficacy of plant protection products. A comparison and evaluation of *Sclerotinia* inoculation techniques are summarized in **Table 1**.

**Table 1:** Comparison of inoculation methods for studying *S. sclerotiorum* pathogenesis and assessing Sclerotinia stem rot resistance.

Inoculum	Preparation of inoculum (days)	Inoculum Preparation Simplicity	Suitability for mass inoculation	Inoculation technique	Target plant organ	Growth stage	Aim of inoculatiom		Selected Reference
							Resistance screening	Fungicide assays	
Under controlled greenhouse conditions									
Mycelium suspension	5 − 7	Easy	Yes	Cotyledon assay	Cotyledon	BBCH 10	x	−	Garg et al., 2008
				Intact leaf inoculation	Leaf	BBCH 61-69	x	x	Thatcher et al., 2017
PDA plugs	5 − 7	Esay	No	Petiole inoculation	Petiole	BBCH 14-15	x	−	Zhao et al., 2004; Bradley et al., 2006
				Detached leaf assay	Leaf	BBCH 14-15	x	−	Bailey, 1987; Liu et al., 2005
				Detached stem assay	Stem	BBCH 75-85	x	−	Mei et al., 2012
				Intact stem inoculation	Stem	BBCH 61-69	x	−	Li et al., 2004
Under natural field conditions									
Ascospore suspension	40-60	Difficult	No	Spraying suspension	Whole plant	BBCH 61-64 & BBCH 65-68	x	−	Bradley et al., 2006
Infested cereal grains	15-20	Average	Yes	Spreading infested grains	Whole plant	BBCH 61-65	x	x	Zamani-Noor, 2021
PDA plugs	5 − 7	Easy	No	Intact stem inoculation	Stem	BBCH 64-65	x	−	Li et al., 2006
Toothpick-method	7 − 10	Easy/Average	No	Intact stem inoculation	Stem	BBCH 81-85	x	−	Zhao and Meng, 2003

This article reviews a wide range of inoculation methods; however, the diversity of *S. sclerotiorum* isolates should also be considered. An important aspect is the clonality of *S. sclerotiorum* lineages (Carbone et al., 1999), although numerous studies conducted in different countries have demonstrated significant morphological and genetic differences between isolates (Ziman et al., 1999; Sun et al., 2005; Baturo-Cieśniewska et al., 2017; Clarkson et al., 2017; Zamani-Noor et al., 2024b). A widely used approach involves assessing mycelial compatibility, where isolates belonging to distinct mycelial compatibility groups (MCGs) are selected for further analyses (Buchwaldt et al., 2022).

## 7 Conclusion

The observation of sclerotia development, the timing and abundance of apothecia formation, and the synchronization of this process with the flowering of oilseed rape are among the most important factors contributing to successful inoculation. During this period, the canopy becomes densely covered with petals, which further facilitates infection if weather conditions are conducive to disease development. However, inoculation with ascospore showers is associated with the risk of late or uneven apothecia formation under laboratory conditions. Despite the advantages of using ascospores for artificial plant infection, this approach is relatively uncommon due to technical difficulties and the extended time required for inoculum preparation. Experimental studies cannot afford the risk of failure, as repeating an experiment would require waiting an entire year until the next growing season. Therefore, developing a standardized protocol in the near future is essential to ensure efficient ascospore production, viable inoculum storage, and successful use in inoculation, thereby reducing unnecessary risks.

Inoculation requires the use of classical phytopathological techniques. Although it seems less technically advanced than modern molecular techniques, it is in fact the crucial tool for assessing the actual resistance of plant genotypes. The skillful using of inoculation methods still poses a challenge in terms of properly selecting agronomic technologies, suitable equipment and manual skills.
